# Nitric Oxide Modulates the Temporal Properties of the Glutamate Response in Type 4 OFF Bipolar Cells

**DOI:** 10.1371/journal.pone.0114330

**Published:** 2014-12-02

**Authors:** Alex H. Vielma, Adolfo Agurto, Joaquín Valdés, Adrián G. Palacios, Oliver Schmachtenberg

**Affiliations:** Centro Interdisciplinario de Neurociencia de Valparaíso, Facultad de Ciencias, Universidad de Valparaíso, Valparaíso, Chile; Dalhousie University, Canada

## Abstract

Nitric oxide (NO) is involved in retinal signal processing, but its cellular actions are only partly understood. An established source of retinal NO are NOACs, a group of nNOS-expressing amacrine cells which signal onto bipolar, other amacrine and ganglion cells in the inner plexiform layer. Here, we report that NO regulates glutamate responses in morphologically and electrophysiologically identified type 4 OFF cone bipolar cells through activation of the soluble guanylyl cyclase-cGMP-PKG pathway. The glutamate response of these cells consists of two components, a fast phasic current sensitive to kainate receptor agonists, and a secondary component with slow kinetics, inhibited by AMPA receptor antagonists. NO shortened the duration of the AMPA receptor-dependent component of the glutamate response, while the kainate receptor-dependent component remained unchanged. Application of 8-Br-cGMP mimicked this effect, while inhibition of soluble guanylate cyclase or protein kinase G prevented it, supporting a mechanism involving a cGMP signaling pathway. Notably, perfusion with a NOS-inhibitor prolonged the duration of the glutamate response, while the NO precursor L-arginine shortened it, in agreement with a modulation by endogenous NO. Furthermore, NO accelerated the response recovery during repeated stimulation of type 4 cone bipolar cells, suggesting that the temporal response properties of this OFF bipolar cell type are regulated by NO. These results reveal a novel cellular mechanism of NO signaling in the retina, and represent the first functional evidence of NO modulating OFF cone bipolar cells.

## Introduction

Nitric oxide (NO) is a volatile, freely diffusible molecule that exerts multiple functions within the central nervous system [1]. Under physiological conditions, the canonical NO receptor is the enzyme soluble guanylate cyclase (sGC), whose activation causes an increase of intracellular cyclic GMP (cGMP) [2]. Subsequently, cGMP may directly activate cyclic nucleotide-gated channels, depolarizing the cells by influx of sodium and calcium ions [3], or activate cGMP-dependent protein kinase (PKG), which phosphorylates diverse proteins [4]. An alternative signaling pathway of NO involves the S-nitrosylation of cysteine residues on specific target proteins [5]. In the retina, NO is synthesized among others by a subset of amacrine cells (AC) termed NI, NII and displaced NOACs [6], [7] and has been shown to amplify the flash electroretinogram in rat [8], but at the cellular level, its effects on retinal signal processing are only partially understood [9]. Some of the reported actions of NO include an increase of light responses in rod bipolar cells (RBC) [10], the modulation of bipolar cell (BC) output through protein S-nitrosylation in goldfish [11], the inhibition of electrical coupling between AII ACs and ON CBCs [12], the regulation of GABA and glycine release from ACs [13], and the activation of cyclic nucleotide-gated channels in retinal ganglion cells (GC) [14]. In addition, NO has been shown to differentially modulate GC ON and OFF responses, which is reflected by a decrease in ON spike responses by about 40%, while OFF responses are abolished completely [15]. However, it remains unclear at which level of retinal processing NO exerts this effect on the OFF response. In turtle, previous evidence indicates that NO increases cGMP levels in OFF CBCs [16], but to date no study has addressed the putative regulation of OFF CBC signaling by NO in mammals. The aim of the present study was to analyze the cellular mechanism by which NO affects the retinal OFF pathway in BCs, using patch clamp recordings and pharmacological stimulation of OFF CBCs. We investigated a putative NO action on OFF BCs selecting the type 4 CBC, because this cell type is easily distinguished from other OFF BC types in rat by its characteristic morphology, since it is the only BC whose axonal endings span both substrata 1 and 2 of the IPL.

Recent reviews have summarized the anatomical and functional characteristics of retinal BCs, and shown that each cell type has specific properties that serve to encode information regarding polarity, time course, intensity and spectral identity of the visual stimulus in a distinct signaling channel [17], [18]. OFF CBCs express sign-conserving ionotropic glutamate receptors in their dendrites [19], [20] and relay complex phasic-tonic responses to GCs [21]. Initial response characteristics of OFF CBCs are shaped by the composition of dendritic glutamate receptors, and mouse type 4 CBCs have been shown to express both the GluA1 and GluK1 subunits of AMPA and kainate receptors, respectively [22]. A similar expression pattern of glutamate receptors in type 4 CBCs can be expected in rat, but needs to be confirmed. Here, we tested the hypothesis that retrograde NO signaling from NOACs affects the glutamate response of rat type 4 CBCs. To this end, type 4 CBCs in rat retinal slices were stimulated with glutamate in the outer plexiform layer (OPL), while superfusing their axonal endings in the inner plexiform layer (IPL) with the NO donor NOC-12. Our results suggest that the temporal properties of the glutamate response in type 4 CBCs are modulated by endogenous NO, acting through the stimulation of sGC, cGMP synthesis and activation of PKG.

## Materials and Methods

### Animals

All experiments were performed on 3–4 week-old Sprague Dawley rats irrespective of sex or weight. The rats, born and raised in the animal facility of the University of Valparaiso (Animal Welfare Assurance NIH A5823-01), were held at 20–30°C under a 12 h photoperiod with water and food *ad libitum*. The experimental procedures were approved by the bioethics committee of the University of Valparaiso, in accordance with the bioethics regulation of the Chilean Research Council (CONICYT) and complied with the ARVO Statement for the Use of Animals in Ophthalmic and Vision Research.

### Retinal slice preparation

Rats were anesthetized by halothane (Sigma, St. Louis, MO, USA) inhalation and sacrificed by decapitation. Eyes were quickly removed and submerged in extracellular solution containing (in mM): 119 NaCl, 23 NaHCO_3_, 1.25 NaH_2_PO_4_, 2.5 KCl, 2.5 CaCl_2_, 1.5 MgSO_4_, 20 glucose and 2 sodium pyruvate. The solution was continuously aerated with 95% O_2_ and 5% CO_2_, reaching a pH of 7.4. Eyes were enucleated and the retina was carefully separated from the sclera. A small piece of retina was embedded in type VII agarose (Sigma) dissolved in a solution containing (in mM): 119 NaCl, 24 HEPES, 1.25 NaH_2_PO_4_, 2.5 KCl, 2.5 CaCl_2_, 1.5 MgSO_4_, pH 7.4, and was glued to the vibratome stage. Retinal slices of 200 µm thickness were made with a vibrating blade microtome (VT1000S, Leica Microsystems, Nussloch, Germany) and maintained in a chamber with oxygenated extracellular solution at room temperature (20°C) and photopic background illumination (100 lux). Retinal slices were then transferred to the recording chamber, held by a U-shaped platinum wire, and superfused with oxygenated extracellular solution at a rate of 1 ml/min, controlled by a peristaltic pump (Masterflex C/L, Cole-Parmer Instruments, Illinois, USA).

### Electrophysiology and stimulation

**Figure 1:**
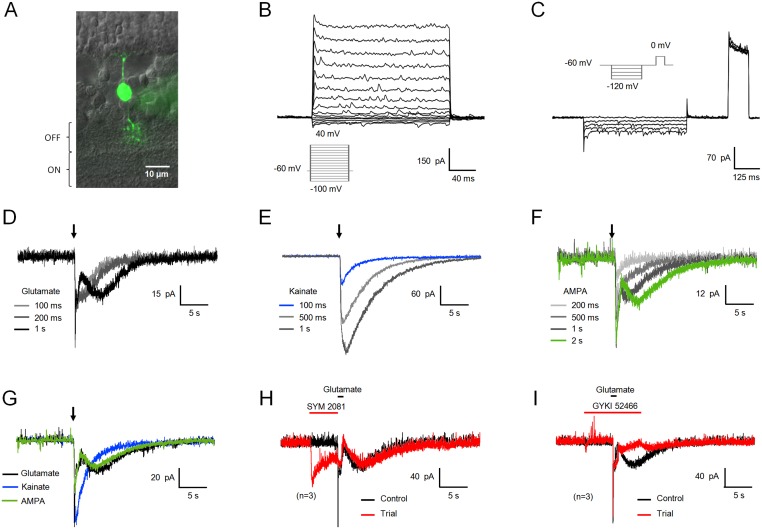
The glutamate response of type 4 CBCs consists of a fast, kainate receptor-, and a slow, AMPA receptor-dependent component. (A) Type 4 CBCs were identified by morphology, notably the position of their axonal endings in substrata 1 and 2 of the IPL, and by their characteristic voltage-gated currents (B, C). These cells lack hyperpolarization-activated cyclic nucleotide-gated (HCN) currents [23]. (D) Glutamate stimuli (500 µM), applied as puffs to the OPL, triggered biphasic inward currents at –60 mV, consisting of a fast peak followed by a slowly decaying tail. At longer stimulus durations, the response current becomes separated into a fast initial peak and a slow secondary component, carrying the bulk of the transferred charge. (E) Kainate stimuli (100 µM) of increasing durations triggered monophasic responses of larger amplitude and longer duration, without development of a separate slow response component. (F) AMPA (300 µM), which activates both AMPA and kainate receptors [32], elicits a two-component response at longer stimulus durations, similar to glutamate. The fast component is present at all stimulus durations. (G) At concentrations adjusted for comparable electrical charge transfer, glutamate (1 s,) and AMPA (2 s,) triggered similar biphasic responses, contrary to kainate (100 ms). (H) The kainate receptor-selective agonist SYM 2081 (50 µM) [63] abolished the fast component of the glutamate response in type 4 CBCs, while the slow component remained unaffected. (I) GYKI 52466 (30 µM), a selective AMPA receptor antagonist [64], blocked the slow component of the glutamate response, without affecting the fast, kainate receptor-dependent component.

Patch clamp recordings were made from type 4 OFF CBCs visualized with an Olympus microscope (BX51WI, Olympus, Japan) equipped with a 40x water-immersion objective, infrared differential interference contrast and a cooled CCD camera (DS-2MBWc, Nikon, Japan) for brightfield and fluorescence image capture. Type 4 OFF CBCs were identified by their characteristic voltage-dependent currents, a conspicuous inhibitory basal activity and lack of I_h_ currents present in other OFF CBC types [23] (Figure 1A–C). Dialysis of Lucifer yellow through the patch pipette allowed us to observe axon terminal stratification of type 4 CBCs within the OFF sublamina of the IPL, to confirm the cell identity. Cells were voltage clamped at –60 mV in the whole-cell configuration. Signals were amplified with an EPC7-plus amplifier (HEKA Elektronik, Lambrecht, Germany), filtered at 3 kHz, digitized and sampled at 10 kHz with an A/D board (PCI-6221, National Instruments, Austin, TX, USA) and recorded using custom-made software written in IGOR PRO (Wavemetrics, Lake Oswego, OR, USA). Electrodes were fabricated using borosilicate glass capillaries (1,5 mm OD, 0,84 mm ID, WPI, Sarasota, FL, USA) and pulled to resistances between 10–15 MΩ on a Flaming/Brown electrode puller (Sutter P-97, Sutter Instruments, Novato, CA, USA). Recording electrodes were filled with an internal solution composed of (in mM): 125 potassium gluconate, 10 KCl, 10 HEPES, 2 EGTA, 2 Na_2_ATP, 2 NaGTP, and 1% Lucifer yellow. pH was adjusted to 7.4 with KOH. To investigate the function of kainate and α-amino-3-hydroxy-5-methyl-4-isoxazolepropionic acid (AMPA) receptors on the dendrites of type 4 CBCs, these agonists were applied at a concentration of 100 µM and 300 µM, respectively. For experiments involving NO, cells voltage-clamped to –60 mV were stimulated with a puff (1 s) of glutamate (500 µM, Sigma) to the OPL, applied from a single-barrel glass pipette with a 1 µm inner tip diameter, using a custom-made picospritzer operating at 2–3 psi of pressure. Before, during and after the glutamate puff, the axonal arbors in the IPL were superfused with a long puff (10 s) of the NO donor NOC-12 (200 µM, Calbiochem), the NO synthesis precursor L-arginine (1 mM, Sigma), or the cGMP analogue 8-bromo-cGMP (8-Br-cGMP, 1 mM, Sigma), applied from single or triple-barrel glass pipettes. The AMPA and kainate receptor blockers GYKI 52466 (30 µM, Tocris Bioscience) and SYM 2081 (50 µM, Tocris), were applied from glass puffer pipettes directed at the OPL. For some experiments, the sGC inhibitor ODQ (100 µM, Sigma) or the PKG inhibitor KT-5823 (1 µM, Sigma) were added to the internal solution of the recording pipette. The NOS-inhibitor 7-NI (100 µM, Calbiochem) as well as the GABA and glycine receptor blockers TPMPA (50 µM, Tocris), SR 95531 (10 µM, Tocris) and strychnine (2 µM, Tocris) were added to the bath solution. NOC-12 was dissolved in 1 M NaOH, while ODQ, KT-5823, GYKI 52466 and 7-NI were dissolved in DMSO and stored at –80°C. All other drugs were dissolved in water except for glutamate, which was prepared as stock solution in PBS.

### nNOS immunohistochemistry

To confirm nNOS expression in NOACs and the IPL, retinal slices were fixed for 20 minutes in 4% paraformaldehyde in PBS. The sections were washed two times for 5 minutes in PBS and then blocked for 1 hour with a solution containing: 1% BSA, 1% horse serum and 0.3% Triton X-100 in PBS, pH 7.4. The primary anti-nNOS antibody (Invitrogen, Santiago, Chile) was diluted 1∶250 in the blocking solution and applied overnight at 4°C. The secondary antibody, donkey anti-rabbit Cy3 (Jackson ImmunoResearch) was diluted 1∶800 in PBS and applied for 1 hour at room temperature. Finally, the slices were rinsed and mounted on microscope slides for inspection and imaging on a confocal microscope (Nikon C1plus, Japan).

### Data analysis and statistics

The maximum amplitude of the glutamate response was measured at the peak of the fast component compared to baseline, using IgorPro. The total charge transfer of the glutamate response was calculated by integrating the area under the curve by a trapezoidal method using Origin Pro Software (www.originlab.com). Quantitative results are shown as bar graphs indicating the mean ± SEM, and statistical significance was evaluated using paired two-tailed Students t-test, using Graph Pad InStat software (La Jolla, CA, USA). In the figures, * and **indicate a significance levels of p<0.05 and p<0.01 respectively.

## Results

### Type 4 OFF CBC glutamate responses display two distinct components

To date, four to five types of OFF CBCs have been described in rodent retina [20], [24], [25]. The light response of each type presents distinct temporal properties, which is reflected in the signal transmitted to GC, as elegantly analyzed recently by 2-photon imaging of calcium signals in their axon terminals [21] and of the responses elicited by OFF CBCs in postsynaptic amacrine and GC dendrites [26]. While it has been reported that the glutamate response of OFF CBCs depends on the activation of AMPA and kainate receptors [19], [27], the kinetic response properties are defined by the combination of different subunits of these receptor classes [22], [28]. Unlike most OFF CBC responses shown to date, which generally exhibit fast (millisecond) kinetics [19], [29], [30], [31], we found that the glutamate response of type 4 CBCs consists of two components, a fast phasic component sensitive to kainate receptor agonists, and a second component with slow kinetics, inhibited by AMPA receptor antagonists. The fast component of the glutamate response could be elicited by short (100 ms and 200 ms) stimuli of glutamate, kainate and also AMPA (Figure 1D–G, n = 3), which has been shown to activate both types of receptors [32]. On the other hand, the distinct slow component of the glutamate response was prominent only at longer glutamate or AMPA stimulus durations (>200 ms), suggesting an elevated response threshold compared to the fast, kainate receptor-dependent component (Figure 1D, F, G). The contribution of distinct receptor types to the biphasic glutamate response was confirmed by application of selective kainate (SYM 2081) and AMPA (GYKI 52466) receptor blockers, which only abolished the fast or the slow component of the glutamate response, respectively (Figure 1H, n = 3; I, n = 3). In summary, glutamate triggers a biphasic response in type 4 CBCs, whose secondary, slow component depends on AMPA receptor activation.

### NO modulates the duration of the AMPA receptor-dependent component of the glutamate response in type 4 CBCs

**Figure 2:**
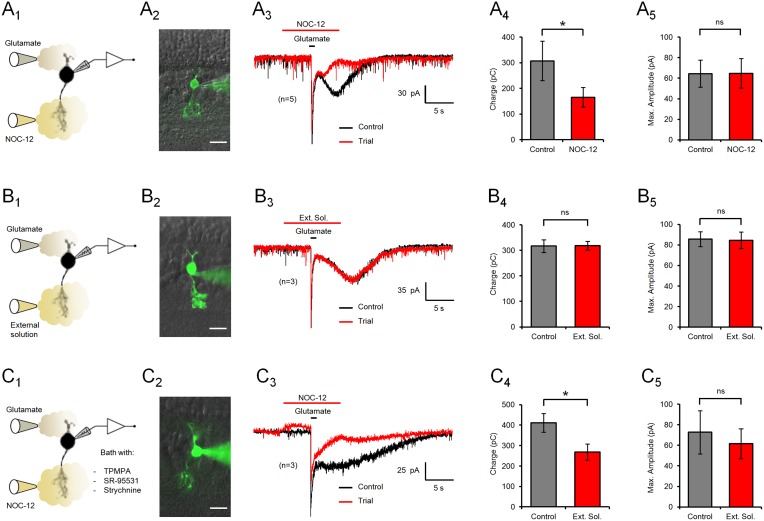
NO modulation of glutamate responses in type 4 CBCs. (A) Representative recordings of glutamate responses of a type 4 OFF CBC, clamped to –60 mV. The experimental setup (A_1_) and an image of the lucifer yellow-filled recorded cell (A_2_) are shown to the left. (A_3_) Application of NO donor NOC-12 (200 µM) only affected the slow component of the glutamate response, by shortening the duration of the electrical response. Bars indicate the stimulus duration. (A_4_) Bar diagrams displaying the mean ± SEM of the total charge transferred during the glutamate response, with and without NO stimulation. (A_5_) The maximum amplitude of the glutamate response, measured at the peak of the fast component, remained unaffected by NO. (B) Control experiments with puffs of extracellular solution instead of NOC-12 were ineffective, demonstrating the absence of stimulus or pressure artifacts. (C) Bath application of the GABA_A_ and GABA_C_ receptor antagonists SR-95531 and TPMPA, and the glycine receptor blocker strychnine did not affect the modulation of the glutamate response by NO in type 4 CBCs. Image scale bars = 10 µm; ns = not significant.

To analyze NO modulation of glutamate responses in type 4 CBCs, the cells were voltage clamped at –60 mV and recorded under light adapted conditions, in absence or presence of the NO donor NOC-12 (Figure 2). Application of NOC-12 caused a prominent change in the temporal properties of the glutamate response, reducing the duration of the slow AMPA receptor-dependent component of the electrical response (Figure 2A_3_). In the presence of this NO donor, electrical charge transferred during the complete response was reduced by 46.1% compared to control conditions (307±77 to 166±38 pC; n = 5; p = 0.03) (Figure 2A_4_), while the amplitude of the glutamate response remained unchanged (64±13 to 65±14 pA; n = 5; p = 0.92) (Figure 2A_5_). These results indicate that NO selectively modulates the slow component of the glutamate response. To rule out stimulus artifacts, the NO donor was replaced by extracellular solution in the stimulus puffs, which did not have any significant effect on the fast (86±7 to 85±8 pA; n = 3; p = 0.47) or the slow component (317±25 to 319±17 pA; n = 3; p = 0.9) of the glutamate response (Figure 2B).

It has been widely reported that NO participates in the regulation of inhibitory signaling in the retina [9]. To verify if NO modulation of the glutamate response involves an inhibitory feedback from horizontal or amacrine cells, glutamate and the NO donor NOC-12 were applied under bath perfusion with GABA_A_, GABA_C_ and glycine receptor blockers (Figure 2C). However, under these conditions, the glutamate response remained sensitive to NO (412±46 to 268±40 pC; n = 3; p = 0.04), suggesting that inhibitory synaptic transmission is not involved in the NO modulation of the glutamate response in CBC 4 cells.

### The effect of NO on type 4 CBC responses depends on cGMP

**Figure 3:**
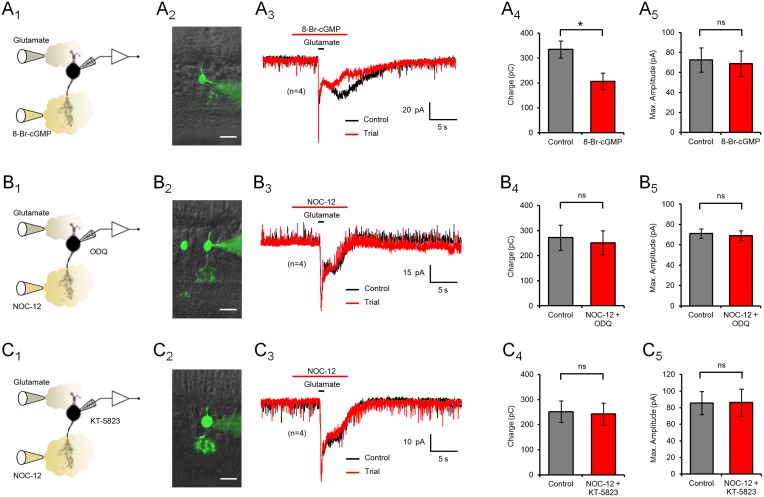
The effect of NO on the slow component of the glutamate response depends on cGMP. (A) Application of the cGMP analog 8-Br-cGMP (1 mM) to the axonal endings of type 4 CBCs (A_1_) reduced the duration of the slow component of the glutamate response (A_3_). (A_4_, A_5_) While the charge transferred during the response was significantly reduced by 8-Br-cGMP, this cGMP analog did not affect the maximum amplitude of the glutamate response. (B) Addition of the sGC inhibitor ODQ (100 µM) to the internal solution of the recording pipette prevented the action of NO on the slow component of the glutamate response in type 4 CBCs (B_3_). Note the previously recorded ON CBC to the left (B_2_). (B_4_, B_5_) Neither the charge transferred during the glutamate response nor the maximum response amplitude were significantly affected by NOC-12 application in presence of ODQ. (C) Similar to ODQ, addition of the PKG inhibitor KT-5823 (1 µM) to the internal solution abolished any effect of NO on the slow component of the glutamate response (C_3_, C_4_). Note that dialysis of ODQ and KT-5823 had a shortening effect on the slow component of the glutamate response by itself. Image scale bars = 10 µm; ns = not significant.

The canonical receptor for NO under physiological conditions is the enzyme sGC, whose activation causes an increase of intracellular cGMP levels [1], [2]. However, reactive nitrogen species derived from NO may also modify cysteine and tyrosine residues, affecting the function of a variety of target proteins [33], [34]. To analyze if the effect of NOC-12 on the slow component of the glutamate response involved cGMP signaling, the permeable cGMP-analog 8-Br-cGMP was applied to the IPL (Figure 3A). 8-Br-cGMP reduced the electrical charge transferred during the glutamate response by 38.2% compared to controls (335±34 to 207±33 pC, Figure 3A_4_), a significant change (n = 4; p = 0.016) that mimics the effect of exogenous NO. Again, the fast glutamate response component remained unchanged (73±12 to 69±13 pA; n = 4; p = 0.09) (Figure 3A_5_). To verify if the effect of NO involves activation of sGC in type 4 CBCs, ODQ, a potent and specific inhibitor of this enzyme, was added to the internal solution of the recording pipette (Figure 3B). Indeed, presence of ODQ in the intracellular solution prevented the action of NO on the slow component of the glutamate response (272±50 to 251±48 pC; n = 4; p = 0.32). This supports the notion that the mechanism by which NO inhibits the slow component of the glutamate response is mediated by activation of intracellular sGC in type 4 OFF CBCs and dependent on cGMP synthesis. To test if the classical cGMP target PKG was also involved in the NO effect, patch clamped type 4 CBCs were dialyzed with KT-5823, a selective inhibitor of this enzyme (Figure 3C). As with ODQ, the reduction of the duration of the slow component of the glutamate response by NO was prevented by this drug (252±43 to 242±44 pC; n = 4; p = 0.074). Interestingly, both ODQ and KT-5823 in the intracellular solution had a shortening effect on the glutamate response independent from NO (Figure 3B_3_ and 3C_3_, respectively), suggesting alternative intracellular mechanisms regulating the slow, AMPA receptor-dependent component of the glutamate response.

### Endogenous NO modulates the slow component of the glutamate response

**Figure 4:**
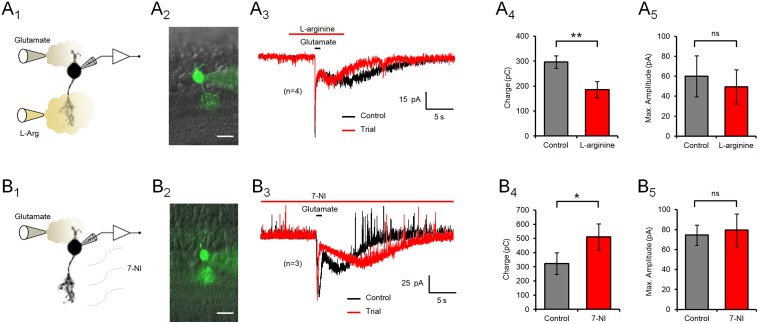
Endogenous NO modulates the time-course of the slow component of the glutamate response. (A) Perfusion of the axonal terminals of type 4 CBCs with the NO precursor L-arginine (1 mM) significantly shortened the slow component of the glutamate response (A_3_) and reduced the overall charge transferred during the response (A_4_), while the maximum response amplitude was unaffected (A_5_). (B) On the other hand, bath perfusion of retinal slices with the NO synthase inhibitor 7-NI (100 µM) significantly increased the charge transferred during the response (B_4_), but did not have an effect on the maximum response amplitude (B_5_) in type 4 CBCs. Image scale bars = 10 µm; ns = not significant.

To investigate the contribution of endogenous NO synthesis to the modulation of glutamate responses in type 4 CBCs, the NO-precursor L-arginine (1 mM) was applied by local perfusion to the IPL (Figure 4A). L-arginine, presumably elevating endogenous NO synthesis, shortened the duration of the slow component of the glutamate response, reducing the charge transferred during the response by 37% (297±25 to 186±33 pC; n = 4; p = 0.003). On the other hand, the NOS inhibitor 7-NI was applied via bath perfusion to retinal slices (Figure 4B). As opposed to the effect of NO donors, application of 7-NI caused an increase in the duration of the slow component of the glutamate response in type 4 CBCs, significantly amplifying the electrical charge transferred during the response by 58% (323±77 to 511±93 pC; n = 3; p = 0.036). These data support the hypothesis that endogenous local NO synthesis modulates the slow component of the glutamate response in type 4 OFF CBCs in the same way as exogenous NO applied through NO donors.

### The time course of the response recovery during repetitive stimulation in type 4 CBCs is controlled by NO

**Figure 5:**
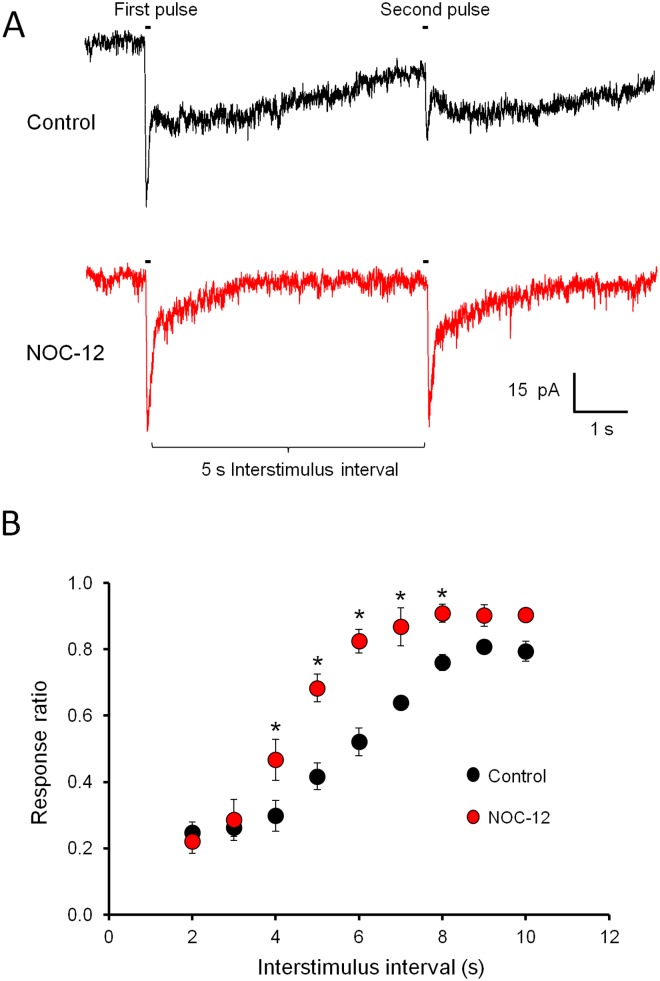
Response recovery during repetitive stimulation is accelerated by NO. (A) Comparison of the recovery of the amplitude of the fast, kainate receptor-dependent component of the glutamate response after two consecutive stimuli (100 ms) with a 5 s interstimulus interval. While reducing the duration of the slow component of the glutamate response, NOC-12 strongly amplified the fast response to the second glutamate stimulus. (B) Amplitude changes of the fast component of the glutamate response at different inter-stimulus intervals in absence and presence of NO donor. The recovery of the kainate receptor-dependent response is significantly accelerated by exogenous NO at intermediate interstimulus intervals (4–8 s).

To understand the physiological significance of NO modulation of the glutamate response in type 4 CBCs, its effect on the response recovery during repetitive stimulation was evaluated. To this end, consecutive glutamate stimuli (100 ms) were applied at distinct interstimulus intervals (2–10 s). At shorter intervals (2–5 s), the amplitude of the fast, kainate receptor-dependent component of the glutamate response decreased by up to 75% (Figure 5), recovering fully only after an interstimulus interval of 10 s. To analyze if NO modulation affects the recovery of the glutamate response, NOC-12 was applied together with glutamate stimuli at different interstimulus intervals (Figure 5B). Indeed, the response recovery during repetitive stimulation was accelerated by NO (n = 3), producing a statistically significant increase of the amplitude of the fast glutamate response component at intermediate interstimulus intervals (4–8 s). These data suggest that NO modulates glutamate response adaptation in type 4 OFF CBCs.

## Discussion

This study presents a novel modulatory mechanism of glutamate responses by NO in a specific OFF CBC type. Type 4 CBC was first identified by morphological characterization [20], [35], and later its immunohistochemical [36], [37], [38], [39] and light response characteristics [21], [40] were described. Studies of the glutamate response in OFF CBCs of rodents revealed the expression and function of dendritic AMPA and kainate receptors in these cells, which do however not co-localize at the cone pedicle base [20], [22], [41]. CBC AMPA receptors are commonly considered “faster” than kainate receptors due to a quicker recovery from desensitization, mediating a more phasic response [19]. Here, we show that the glutamate response of rat CBC 4 consists of two components, a fast and a distinct slow component. The first has similar kinetics to classical OFF CBC responses reported in other studies [29], [31]. The slow component has not been specifically identified previously to our knowledge, although new evidence indicates that type 4 CBCs have slower response kinetics than other BCs [21], [22]. While a response with a time course of several seconds can hardly contribute directly to image formation in the retina, it is conceivable that it represents an adaptive mechanism responding to different levels of glutamate concentration at the photoreceptor-bipolar cell synapse, setting the threshold for fast, kainate receptor-dependent responses (Figure 5). A recent report revealed the involvement of AMPA receptors in the ground squirrel retina predominantly in type 2 CBCs (nomenclature of this species), suggesting that the bulk of synaptic transmission through the remaining OFF CBC types is mainly carried by kainate receptors [28]. Another recent study in mice suggested that the light response of OFF CBCs may be mediated almost exclusively by kainate receptors [26]. It is therefore possible and warrants further investigation that AMPA receptors of OFF CBCs activate only at higher glutamate concentrations, having a modulatory or light/dark adaptive function rather than a fast signal transmitting role.

It has been reported that type 4 CBCs receive inhibitory signaling mediated by GABA_A_, GABA_C_ and glycine receptors [42], [43], and retinal inhibitory signaling is modulated by NO [13], [44], [45]. However, the possibility that the inhibition of the glutamate response by NO is the result of an inhibitory feedback mediated by GABA or glycine, which may be released by glutamate stimulation of horizontal cells or indirect activation of amacrine cells, is not supported by our data, since blockage of GABA_A_, GABA_C_ and glycine receptors did not abolish the effect (Figure 2C).

**Figure 6:**
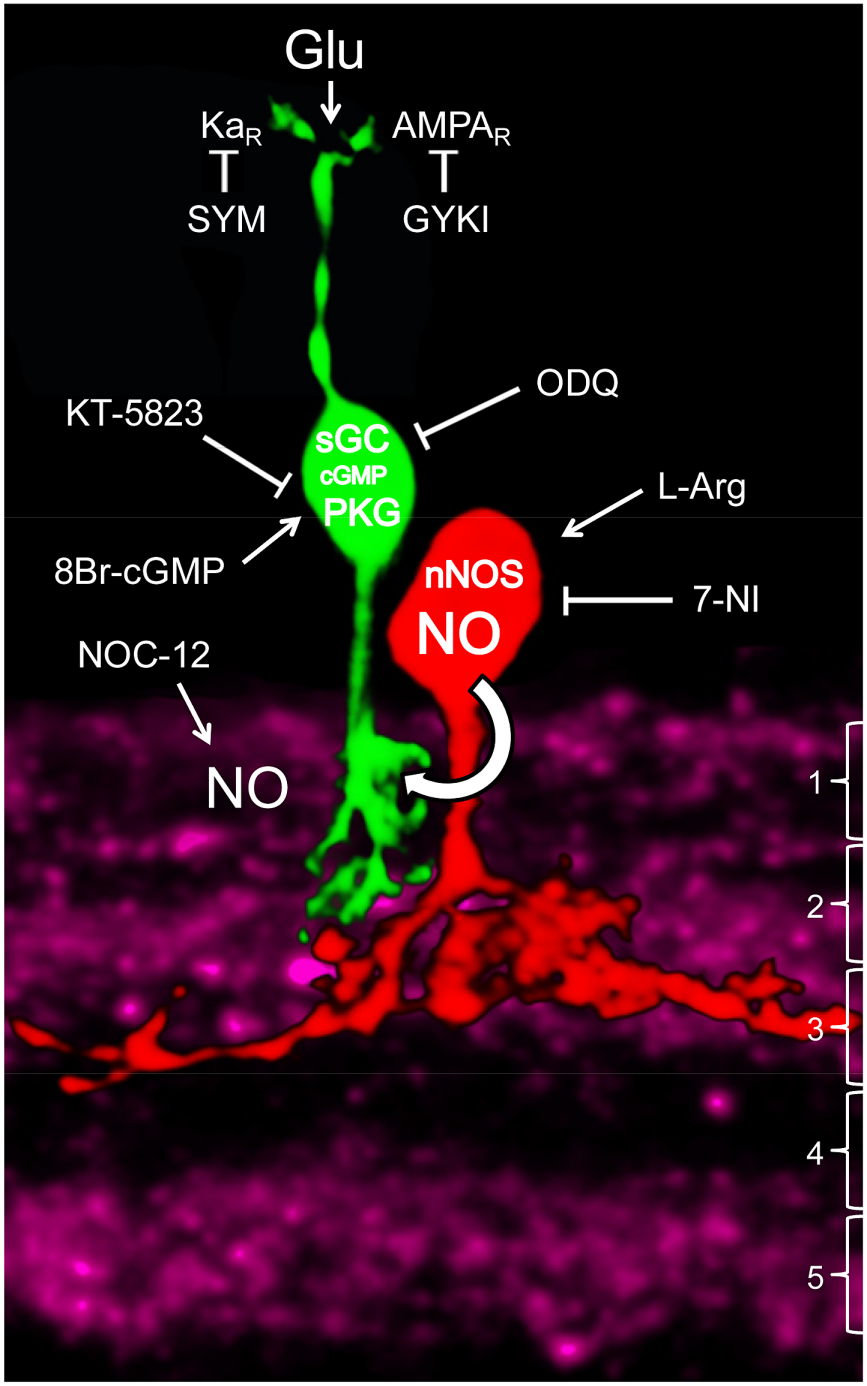
Scheme of proposed NO signaling onto type 4 OFF CBCs in the inner retina. The profiles of a nNOS-positive NOAC (red) and a type 4 OFF CBC (green) are projected onto the nNOS-labeled IPL (purple). The substrata of the IPL are indicated to the right. nNOS is most concentrated in the substrata 2–3 and 5 and largely absent from substratum 4. NOACs mainly ramify in sublayer 3 of the IPL [7]. Glutamate (Glu) from cones or exogenous stimulation activates kainate and AMPA receptors (Ka_R_ and AMPA_R_), whose responses can be blocked by SYM-2081 (SYM) and GYKI-52466 (GYKI), respectively. NO, liberated by NOACs or the NO donor NOC-12, diffuses into CBCs, activating sGC, leading to cGMP elevation which stimulates protein kinase G (PKG). This pathway can be emulated by 8Br-cGMP application and inhibited by ODQ or KT-5823. Endogenous NO synthesis is stimulated by L-arginine and inhibited by 7-NI. The mechanism by which the AMPA-dependent glutamate response is inhibited via PKG remains to be shown.

Few studies have analyzed the cellular basis for the role of NO in the modulation of the retinal OFF pathway [15], [46], but the presence of sGC in OFF CBCs [47] suggests that NO might modulate this pathway at the BC level. However, a direct effect of NO on BC responses has only been reported for RBCs [10]. Our data represent novel evidence for a modulatory role of NO in the inner retina, and support a proposed mechanism for the temporal regulation of OFF CBC responses by NO (Figure 6). To our knowledge, this specific mechanism of glutamate response modulation by NO has not been described in other cells or systems, and similar or identical mechanisms might operate in other circuits of the nervous system.

Activation of sGC in BCs has been shown in several species through an increase of cGMP immunoreactivity after NO donor application [16], [48], [49], [50], [51], [52]. However, only a few studies have specified if the labeled cells correspond to OFF CBCs [16], [49]. Here, we demonstrate that the effect of NO on the slow component of the glutamate response is also dependent on cGMP and can be blocked by addition of the sGC inhibitor ODQ to the intracellular solution. Previous studies reported different aspects of retinal NO signaling, involving pathways sensitive to ODQ, inferring a cGMP-dependent mechanism leading to PKG activation [9], [10], [44], [53], [54]. Our data suggest that in the mechanism described here, NO acts through activation of sGC and subsequent stimulation of PKG in type 4 OFF CBCs, as in the NO signaling pathways proposed by the aforementioned studies. While the established source of NO in the retina are NOACs [6], [7], [55], expression of NO synthase in BC has also been reported in several species [6], [55], [56], [57], [58], [59], [60]. Furthermore, assays with the NO-indicators DAF-2 or DAF-FM indicated that an unidentified and possibly variable subgroup of BCs synthesize NO [46], [57], [61]. Our data are consistent with the idea of endogenous retrograde NO signaling from NOACs to type 4 OFF CBCs (Figure 6), but we cannot exclude the possibility of additional or alternative NO sources among the different BC types [9], including autocrine NO signaling in BCs.

In summary, we describe a system in which NO participates as modulator of temporal properties of the glutamate response, and propose a working model to explain NO regulation of a specific OFF cone BC type, which depends on endogenous synthesis of NO, activation of sGC and production of cGMP. The slow time-course of the glutamate response component regulated by NO makes it an attractive candidate for a light adaptational mechanism within the OFF pathway, in line with mounting evidence that NO is involved in retinal light/dark adaptation [9], [62].
