# Correction: Nitric Oxide Modulates the Temporal Properties of the Glutamate Response in Type 4 OFF Bipolar Cells

**DOI:** 10.1371/journal.pone.0119399

**Published:** 2015-03-23

**Authors:** The PLOS ONE Staff

Fig. 2 is incorrect. The authors have provided a corrected version here.

**Fig 2:**
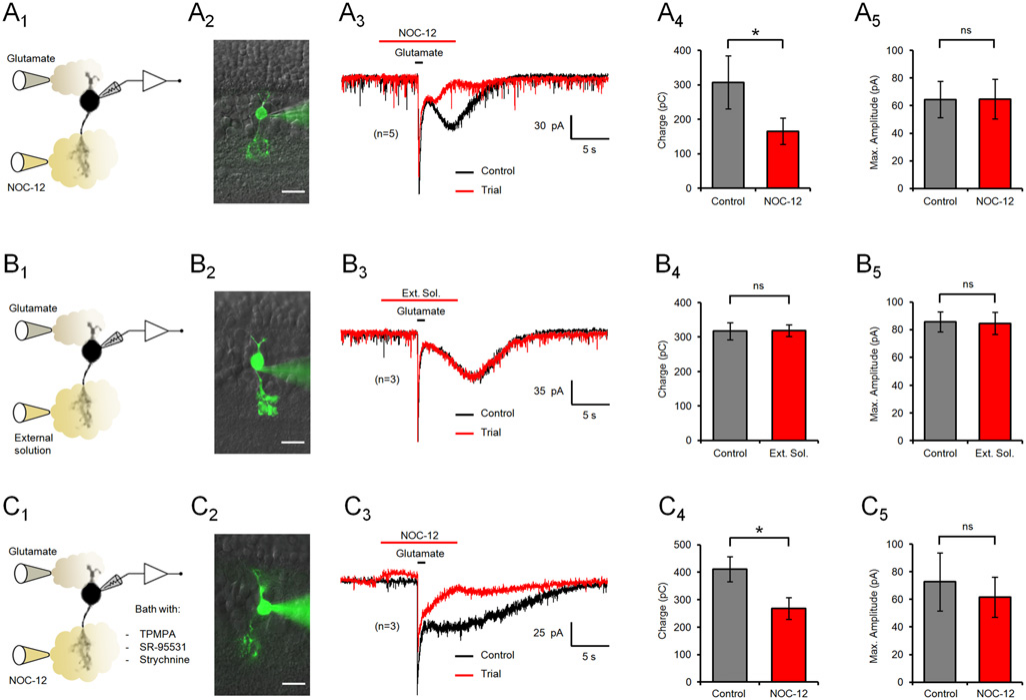
NO modulation of glutamate responses in type 4 CBCs. (A) Representative recordings of glutamate responses of a type 4 OFF CBC, clamped to –60 mV. The experimental setup (A_1_) and an image of the lucifer yellow-filled recorded cell (A_2_) are shown to the left. (A_3_) Application of NO donor NOC-12 (200 μM) only affected the slow component of the glutamate response, by shortening the duration of the electrical response. Bars indicate the stimulus duration. (A_4_) Bar diagrams displaying the mean ± SEM of the total charge transferred during the glutamate response, with and without NO stimulation. (A_5_) The maximum amplitude of the glutamate response, measured at the peak of the fast component, remained unaffected by NO. (B) Control experiments with puffs of extracellular solution instead of NOC-12 were ineffective, demonstrating the absence of stimulus or pressure artifacts. (C) Bath application of the GABA_A_ and GABA_C_ receptor antagonists SR-95531 and TPMPA, and the glycine receptor blocker strychnine did not affect the modulation of the glutamate response by NO in type 4 CBCs. Image scale bars = 10 μm; ns = not significant.
